# Non-Fickian Moisture Absorption in Vegetable Fiber Reinforced Polymer Composites: The Effect of the Mass Diffusivity

**DOI:** 10.3390/polym13050761

**Published:** 2021-02-28

**Authors:** Rafaela Q. C. Melo, Marcus V. Lia Fook, Antonio G. B. Lima

**Affiliations:** 1 Department of Materials Engineering, Federal University of Campina Grande (UFCG), Campina Grande, 58429-900, Paraiba, Brazil; marcus.liafook@certbio.ufcg.edu.br; 2 Department of Mechanical Engineering, Federal University of Campina Grande (UFCG), Campina Grande, 58429-900, Paraiba, Brazil; antonio.gilson@ufcg.edu.br

**Keywords:** vegetable fibers, Langmuir-type model, water absorption, variable mass diffusivity, numerical simulation

## Abstract

This article aims to study the non-Fickian water absorption process in vegetable fiber-reinforced polymer composite using the Langmuir-type model, evaluating the influence of mass diffusivity on the process. The numerical solutions of the governing equations were obtained using the finite-volume method. Transient results of the local and average moisture content, free and entrapped water molecules concentration considering the constant diffusivity and as a function of the average and local moisture content were presented and analyzed. It was observed that the mass diffusivity effectively influences the water absorption behavior, especially in the initial time of the process, where higher differences in the water migration rates into the material are found. The largest free and entrapped water molecule concentration gradients were found close to the composite surface, especially when considering constant mass diffusivity.

## 1. Introduction

Recently, environmental issues have been strongly considered in the context of technological and sustainable development. This fact is evidenced by the growing academic and industrial interest in alternative materials with renewable and biodegradable characteristics, mainly in the polymeric composite materials area. Thus, the use of vegetable fiber as reinforcing to replace synthetic fibers has been presented as a potential substitute, decreasing the pollutants generation and resulting in products with adequate performance and quality that meet the demands of different applications [1,2].

One of the main advantages of the use of vegetable fiber reinforcement in the polymer matrix is its low density, which allows the manufacture of lighter products with specific mechanical properties comparable to conventional fibers [3]. However, despite these positive effects, the hydrophilic nature of the vegetable fibers increases the moisture absorption levels, directly affecting the mechanical properties and composites’ performance in operation [4,5]. Thus, it is important to study the behavior of these materials when exposed to moisture in order to estimate the consequences in their properties and to minimize these effects, especially at higher temperatures.

Due to the importance of this topic, several studies about the water absorption process in vegetable fiber-reinforced polymer composites have been reported in the literature under different operational conditions [6,7,8,9,10]. However, obtaining experimental data takes a long time, in general, some weeks before saturation condition is reached; therefore, in order to obtain faster information about the moisture migration behavior, with low cost, several researchers have studied this physical problem by numerical simulation using developed mathematical models.

Several authors have suggested different models and their solutions to describe the water sorption kinetics in polymer composites [11,12,13,14,15,16,17]; among them, Fick’s diffusion model has been the most used. However, polymer composite during the water absorption process may present anomalous behavior (non-Fickian diffusion), where the moisture uptake initially presents similar behavior to the Fickian diffusion, being faster in the initial times with constant diffusivity and becoming slower with variable diffusivity [18]. Then, it is necessary to use models with new approaches that adequately capture this anomalous behavior during the water absorption process.

Based on the different models reported in the literature, it can be seen that water diffusion can be described considering two stages: first, Fickian diffusion, which occurs by free water molecules available to diffuse inside the material, and second, non-Fickian diffusion, caused by water molecules that become entrapped through chemical bonds in the polymer chains [19]. The phenomena can be explained by the Langmuir-type model [20], which assumes chemical interactions between polar water molecules and the polymer chains. The model modifies Fick’s equation coupling these two stages; thus, the process is described in an improved way, allowing physical interpretations to be more realistic, according to the real physical phenomena that may occur during the water sorption process.

Thus, some researchers have recently used Langmuir’s model to describe the water absorption behavior in vegetable-fiber-reinforced composites. Regazzi et al. [21] investigated the hydrothermal aging of Polylactic acid (PLA)/flax composites at different temperatures using a numerical model for describing the reversible effects involved during the aging process (water diffusion described by Fick and Langmuir models). The simulated results agreed well with experimental data and showed that the diffusion and swelling behaviors were perfectly simulated in the selected conditions. Santos et al. [22] theoretically studied the water sorption process in vegetable-fiber-reinforced polymer composites using Langmuir’s model in a three-dimensional and transient approach based on the finite-volume method. The obtained results for the free and entrapped molecules concentration and local and average moisture content at different process times were presented and analyzed. The simulated data were validated for polyester/sisal composites. The results show a good agreement with experimental data and allow a detailed interpretation of the physical phenomenon. Melo et al. [23] presented a new approach for the Langmuir-type model in a one-dimensional and transient solution in order to evaluate the water layer thickness effect during the absorption process. The results of the free and entrapped water concentration, local and average moisture content, concentration gradients and variation rate of free and entrapped water molecules during the process were presented. The authors observed that the water layer thickness strongly affects the absorption process, allowing a better interpretation with the experimental data and better understanding of the physical phenomenon.

It is important to state that, in many works related to Langmuir’s model, the authors considered constant mass diffusivity [22,23,24,25,26]. However, this assumption is not completely consistent with the real process behavior, since the moisture migration rates are higher at the shorter process times and decrease for longer times; therefore, mass diffusivity varies with the water concentration inside the material [27]. Even among the works that consider variable mass diffusivity (with the general Fick´s model), there are still few studies that established the mass diffusion coefficient as a function of the average moisture content [28,29,30,31,32], and almost none that consider mass diffusion coefficient as a function of the local moisture content inside the material.

Another important fact related to the water sorption experiments in polymer composites reinforced with vegetable fibers is that moisture diffusion occurring in the sample is conditioned by simultaneous moisture fluxes on the different surfaces exposed to water. Thus, the samples, when placed in the water bath, may show differences in water absorption on the upper and lower sides due to the accommodation of the material at the container bottom. Although this phenomenon is perfectly observed, researchers consider that the boundary conditions to be the same at the material surface, contrary to the real process conditions, which can lead to severe errors in the parameters estimation and phenomenon prediction. Thus, considering these observations and those already mentioned in this paper and incorporating them into the mathematical model are of fundamental importance for a detailed understanding of the phenomena involved in the water absorption process, their physical interpretations and implications for the properties and durability of these materials.

In this sense, in compliment to the works reported in the literature, this research aims to evaluate the effect of the mass diffusion coefficient on the water absorption kinetics in vegetable fiber-reinforced polymer composites using an advanced diffusional macroscopic modeling that predicts molecular interactions between water and the composite material, as well as the material position in the water bath. The one-dimensional solution of the governing equations was obtained numerically, using the finite-volume method, considering the constant and variable mass diffusivity and non-deformable material. It is, therefore, an innovative research study in this area.

## 2. Methodology

### 2.1. The Physical Problem and Geometry

**Figure 1:**
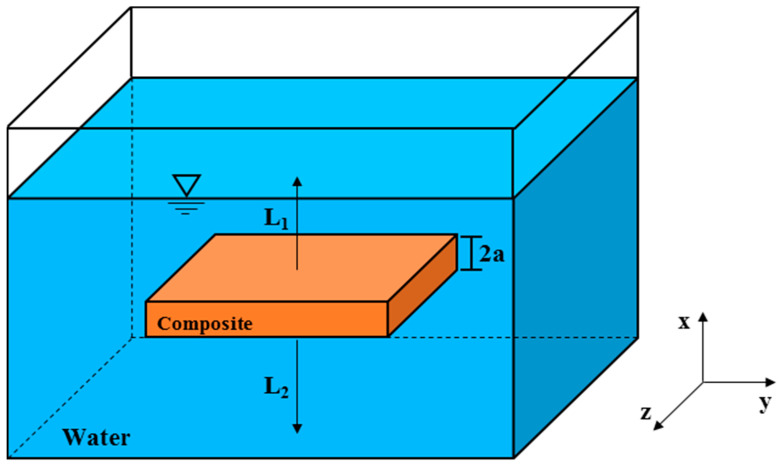
Geometric representation for the proposed physical problem.

The mathematical modeling of the physical problem proposed here considered a solid material (composite) with 2a thickness, immersed in a fluid solute (water) inside a container. The sample is located at a distance L_1_ and L_2_ from the upper and down water levels in the container. The water diffusion process was considered to be one-dimensional and occurring only along the direction x. The physical problem treated here is illustrated in Figure 1.

### 2.2. The Mathematical Model

Langmuir’s model assumes that water absorption inside the material occurs through two stages: in the first stage, the water molecules are free to diffuse throughout the material and with a probability of becoming entrapped in the structure, and the second, the entrapped water molecules have a probability to become again free for diffusion.

The following hypotheses were considered for adequate mathematical modeling: (a) the material is homogeneous and isotropic; (b) the process is transient; (c) the mass transport mechanism inside the material is pure diffusion; (d) the solid is completely dry at the beginning of the process; (e) there is equilibrium condition between the external environment and solid surfaces; (f) the water migration rate leaving the water bath is equal to the water diffusive flux on the composite surface, and (g) there is no water mass generation inside the composite.

Based on the hypotheses cited, Langmuir’s model, in a one-dimensional form and in Cartesian coordinates, is described using the following equations:

(1) $$\frac{\partial C}{\partial t}=\frac{\partial }{\partial x}\left(D\frac{\partial C}{\partial x}\right)-\frac{\partial S}{\partial t}$$

and

(2) $$\frac{\partial S}{\partial t}=\lambda C-\mu S$$

In Equation (1), C and S represent, respectively, the free and entrapped water molecules concentration inside the composite, D is the mass diffusivity of the free water molecules, λ and μ are the probabilities for the free and entrapped phases, and t represents time. Equation (2) represents the source term that describes the temporal variation of the entrapped water molecules concentration inside the material.

For the solution of the governing equations, the following initial and boundary conditions were considered:

(a) Initial condition

(3) $$C=S=0\;\;\{\begin{array}{l} 0<x<2a\\ t=0 \end{array}$$

(b) Boundary condition

(4) $$L_{1}\frac{\partial C}{\partial t}=-D\frac{\partial C}{\partial x}\;\;\{\begin{array}{l} x=2a\\ t>0 \end{array}$$

(5) $$L_{2}\frac{\partial C}{\partial t}=-D\frac{\partial C}{\partial x}\;\;\{\begin{array}{l} x=0\\ t>0 \end{array}$$

where x = 0 represents the bottom position of the container and 2a is the composite thickness, as shown in Figure 1.

Equation (4) represents the water flux at the upper boundary, and Equation (5) represents the same flux at the lower boundary of the composite. The incorporation of both boundary conditions into Langmuir’s model allows the evaluation of the difference between the diffusive fluxes at the composite surfaces and the effects of the water level on the absorption kinetics. Furthermore, in Equation (1), the mass diffusion coefficient can be considered constant or variable throughout the process.

For the analysis of the water sorption kinetics, the total moisture content inside the material is obtained from the sum between the C and S values, using the following equation:

(6) M=C+S

The Equation (6) represents the local moisture content, that is, the moisture content at a specific position x and at an instant of time t. Once the local moisture content is determined, the average moisture is obtained from the volumetric integration of Equation (6) divided by the volume of the solid, given through the following relationship (considering a one-dimensional problem):

(7) $$\bar{M}=\frac{1}{V}\int MdV=\frac{1}{2a}\int_{0}^{2a}Mdx$$

where V represents the volume of the solid.

The mass diffusivity is an important parameter in predicting and studying the characteristics of water absorption in materials. The water penetration inside the material causes changes in its internal structure, and these effects can influence its effective mass diffusivity [33]. Thus, it is crucial to describe the moisture uptake kinetics considering variations in this process parameter.

Thus, the water absorption process was simulated considering the situation with constant mass diffusion coefficient and as a function of the local and average moisture content. The following relationships were considered:

(8) $$D\left(M\right)=D_{o}\exp\left(-\eta M\right)$$

(9) $$D\left(\bar{M}\right)=D_{o}\exp\left(-\eta \bar{M}\right)$$

(10) $$D=D_{o}$$

where Do is the initial mass diffusivity of the free water molecules, η is a constant, M represents the local moisture content and $\bar{M}$ is the average moisture content.

### 2.3. Numerical Solution

To obtain the solutions of the governing equations, the finite-volume numerical method was used. This method consists of transforming the physical domain into a discrete domain, by discretizing the equation from the integration of these equations in volume and time.

**Figure 2:**
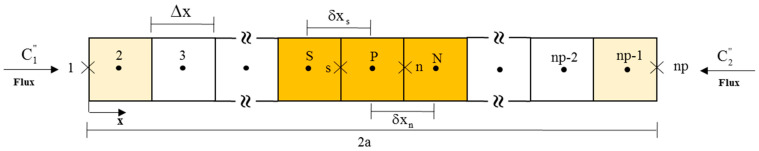
Representation of the discretized physical domain and its respective nodal points.

The water absorption process was analyzed using a one-dimensional approach; thus, the diffusive flux occurs along the x direction. For the discretization process, a solid with uniform thickness 2a and (np-1) control-volumes was considered. Figure 2 schematically illustrates the computational domain, where P is the nodal point, N and S are North and South neighbors, respectively, Δx is the control-volume dimension, and $\delta x_{s}$ and $\delta x_{n}$ are the respective distance between point P and its neighbors.

For analysis of the process, the domain under study was divided into three regions: (a) internal volumes; (b) upper boundary volumes and (c) lower boundary volumes. The solutions were obtained for each distinct region (both for free and entrapped water molecules concentration).

#### 2.3.1. Free Water Molecules Concentration

(a) Internal control volumes (Central points)

For the discretization process, Equation (1) is integrated into the control volume of point P and in time. The values of variables related to the diffusive fluxes in the control-volumes centers are obtained as a function of Taylor´s series expansions around the interface. Thus, for the left control volume (S) and right control volume (N) interfaces, the following relationships for derivatives are used:

(11) $${\frac{\partial C}{\partial x}|}_{n}=\frac{C_{N}-C_{P}}{\delta x_{n}}$$

(12) $${\frac{\partial C}{\partial x}|}_{s}=\frac{C_{P}-C_{S}}{\delta x_{s}}$$

After the integration process, using a fully implicit formulation and considering the diffusive fluxes equations in each of the volumes faces, Equation (1) assumes the following form:

(13) $$\left(C_{P}-C_{P}^{o}\right)\frac{\Delta x}{\Delta t}=D_{n}\left(\frac{C_{N}-C_{P}}{\delta x_{n}}\right)-D_{s}\left(\frac{C_{P}-C_{S}}{\delta x_{s}}\right)-\left(\lambda C_{P}-\mu S_{P}^{o}\right)\Delta x$$

Rearranging the terms of Equation (13) in the discretized linear algebraic form and applied to point P, the following equation (valid for all internal points of the domain) is obtained:

(14) $$A_{P}C_{P}=A_{N}C_{N}+A_{S}C_{S}+A_{P}^{o}C_{P}^{o}+BS_{P}^{o}$$

where

(15) $$A_{P}=\left(\frac{\Delta x}{\Delta t}+\frac{D_{n}}{\delta x_{n}}+\frac{D_{s}}{\delta x_{s}}+\lambda \Delta x\right)$$

(16) $$A_{N}=\left(\frac{D_{n}}{\delta x_{n}}\right)$$

(17) $$A_{S}=\left(\frac{D_{s}}{\delta x_{s}}\right)$$

(18) $$A_{P}^{o}=\left(\frac{\Delta x}{\Delta t}\right)$$

(19) B=(μΔx)

The coefficients A_P_, A_N_ and A_S_ represent the contributions of diffusive transport between point P and its corresponding neighbors. The term $A_{P}^{o}$ represents the influence of the value of variable C in the previous time over its value at the current time.

(b) Upper boundary control volumes

For the upper boundary control-volumes, the discretization process is done in a similar way to Equation (13); however, this case considers the water flux on the left side of the control-volume (face s) and the water flux on the boundary $\left(C_{1}^{\prime \prime }\right)$, which must be replaced according to the boundary conditions suggested in the problem. Thus, from performing the discretization of Equations (1) and (4) and making the appropriate substitutions, the following equation is obtained:

(20) $$\left(C_{P}-C_{P}^{o}\right)\frac{\Delta x}{\Delta t}=-C_{1}^{\prime \prime }-D_{s}\left(\frac{C_{P}-C_{S}}{\delta x_{s}}\right)-\left(\lambda C_{P}-\mu S_{P}^{o}\right)\Delta x$$

where,

(21) $$C_{1}^{\prime \prime }=\left(\frac{\frac{D_{n}}{\delta x_{n}}C_{P}-\frac{D_{n}}{\delta x_{n}}C_{n}^{o}}{1+\frac{D_{n}\Delta t}{\delta x_{n}L_{1}}}\right)$$

Rearranging the terms of Equation (20), the following discretized linear algebraic equation is obtained for the upper boundary points:

(22) $$A_{P}C_{P}=A_{S}C_{S}+A_{n}^{o}C_{n}^{o}+A_{P}^{o}C_{P}^{o}+BS_{P}^{o}$$

where

(23) $$A_{P}=\left(\frac{\Delta x}{\Delta t}+\frac{1}{\frac{\delta x_{n}}{D_{n}}+\frac{\Delta t}{L_{1}}}+\frac{D_{s}}{\delta x_{s}}+\lambda \Delta x\right),$$

(24) $$A_{S}=\left(\frac{D_{s}}{\delta x_{s}}\right)$$

(25) $$A_{n}^{o}=\left(\frac{1}{\frac{\delta x_{n}}{D_{n}}+\frac{\Delta t}{L_{1}}}\right)$$

(26) $$A_{P}^{o}=\left(\frac{\Delta x}{\Delta t}\right)$$

(27) B=(μΔx)

(c) Lower boundary control volumes

For the lower boundary region, the equation is obtained in a similar way to the upper boundary; however, in this case, the flux on the right side of the volumes (face n) and the water flux in the boundary $\left(C_{2}^{\prime \prime }\right)$ are considered, which must be replaced according to the proposed boundary conditions. Thus, from performing the discretization of Equations (1) and (5) and making the necessary substitutions, the following equation is obtained:

(28) $$\left(C_{P}-C_{P}^{o}\right)\frac{\Delta x}{\Delta t}=-C_{2}^{\prime \prime }-D_{s}\left(\frac{C_{P}-C_{S}}{\delta x_{s}}\right)-\left(\lambda C_{P}-\mu S_{P}^{o}\right)\Delta x$$

where

(29) $$C_{2}^{\prime \prime }=\left(\frac{\frac{D_{n}}{\delta x_{n}}C_{P}-\frac{D_{n}}{\delta x_{n}}C_{n}^{o}}{1+\frac{D_{n}\Delta t}{\delta x_{n}L_{2}}}\right)$$

Rearranging the terms of Equation (28), the following discretized linear algebraic equation for the points of the lower boundary is obtained:

(30) $$A_{P}C_{P}=A_{N}C_{N}+A_{S}^{o}C_{S}^{o}+A_{P}^{o}C_{P}^{o}+BS_{P}^{o}$$

where

(31) $$A_{P}=\left(\frac{\Delta x}{\Delta t}+\frac{1}{\frac{\delta x_{s}}{D_{s}}+\frac{\Delta t}{L_{2}}}+\frac{D_{n}}{\delta x_{n}}+\lambda \Delta x\right)$$

(32) $$A_{N}=\left(\frac{D_{n}}{\delta x_{n}}\right)$$

(33) $$A_{S}^{o}=\left(\frac{1}{\frac{\delta x_{s}}{D_{s}}+\frac{\Delta t}{L_{2}}}\right)$$

(34) $$A_{P}^{o}=\left(\frac{\Delta x}{\Delta t}\right)$$

(35) B=(μΔx)

The Equations (14), (22) and (30) applied at each nodal point in the control-volumes form a linear algebraic equations system whose solution results in the free water molecules concentration within the solid during the moisture absorption process.

The effective mass diffusivity at the control-volume interfaces, which appear in Equations (14), (22) and (30), can be estimated by assuming a variation between the values of this parameter in the nodal point P and its neighbors in the north (N) and south (S) directions. Thus, is obtained by finding the harmonic average of the values of this parameter, in the corresponding nodal points, through the following relation:

(36) $$D_{i}=\frac{2D_{a}D_{P}}{D_{a}+D_{P}}$$

where D_P_ refers to water mass diffusivity in the nodal point P and the subscript a is an index referring to the nodal point of the direction under analysis. Equation (36), from a physical point of view, is more effective and realistic; if the mass diffusivity at point P or its neighbors is zero, the water flux will be null, which is consistent with the real phenomena.

#### 2.3.2. Entrapped Water Molecules Concentration

To determine the values of entrapped water molecules concentration, the discretization process was performed in Equation (2), considering a fully explicit formulation, obtaining the following equation:

(37) $$\left(S_{P}-S_{P}^{o}\right)\frac{\Delta x}{\Delta t}=\left(\lambda C_{P}-\mu S_{P}\right)\Delta x$$

Rearranging the terms of Equation (37), the following discretized algebraic linear equation (valid for all points of the control volume) is obtained:

(38) $$A_{P}S_{P}=A_{P}^{o}S_{P}^{o}+B$$

where

(39) $$A_{P}=\left(\frac{\Delta x}{\Delta t}+\mu \Delta x\right)$$

(40) $$A_{P}^{o}=\left(\frac{\Delta x}{\Delta t}\right)$$

(41) $$B=\lambda \Delta xC_{P}$$

#### 2.3.3. Local and Average Moisture Contents

The total moisture inside the material at a specific position x and in an instant of time t is given by the sum of the values of the free and entrapped water molecules concentrations, as described mathematically by Equation (6). Already the average moisture content is obtained from the discretization of Equation (7) and assumes the following format:

(42) $$\bar{M}=\frac{1}{2a}\sum_{i=2}^{np-1}M\Delta x$$

Equations (6) and (42) are valid for all points in the computational domain.

### 2.4. Computational Simulation

To obtain the numerical solution of the discretized equations, a computational code was developed on the *Mathematica ^®^* software (Wolfram, Champaign, Illinois, USA). The linear algebraic equations system generated from the discretization of Equation (1) was solved iteratively using the Gauss-Seidel method, where the following convergence criterion was assumed at each nodal point:

(43) $$\left|C_{P}^{n+1}-C_{P}^{n}\right|\leq 10^{-10}$$

where n represents the nth iteration in each instant of time.

Thus, once the values of C were determined, at any nodal point in the domain, the values of S were easily determined by Equation (38).

After a study of mesh refining and time step, a numerical mesh with 30 nodal points (np = 30) and a time step of 20 s (Δt=20 s) was chosen for simulation, since these values guaranteed a correct prediction of the results associated with a low computational time.

### 2.5. Evaluated Cases

To simulate the water absorption behavior and evaluate the influence of water mass diffusivity on the process, different arbitrary cases were defined, as illustrated in Table 1. The absorption process was simulated considering an equilibrium moisture content Me = 0.14488 kg/kg, until approximately 2250 h (3 months of the process). The values for the parameters used in the simulation were defined based on the results previously reported in the literature [23]. Observe that two physical situations were considered: one where both the sides of the composites are submitted to a water layer of thickness 10 cm, and another where one side of the composite is submitted to a water layer of thickness 1 mm and another face to 10 cm water layer thickness.

## 3. Results and Discussions

Aging from humidity is a major cause of long-term failures in vegetable-fiber-reinforced polymeric composites when exposed to the atmosphere or in contact with aqueous media. In these cases, two main characteristics play an important role in this behavior: the hydrophilic characteristic of the vegetable fibers and the water mass diffusivity inside the material [34]. As water absorption occurs, chemical and physical processes happen in the material, causing changes in its structure and, consequently, decreasing the water penetration rate with increasing immersion time. This proves that the water mass diffusivity is predominantly variable throughout the process and not constant, as used by different researchers [35].

**Figure 3:**
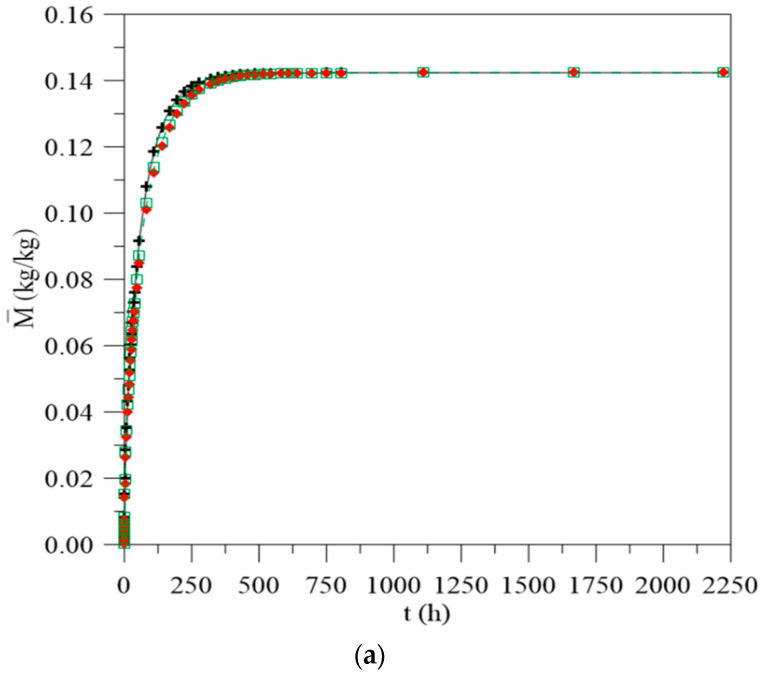

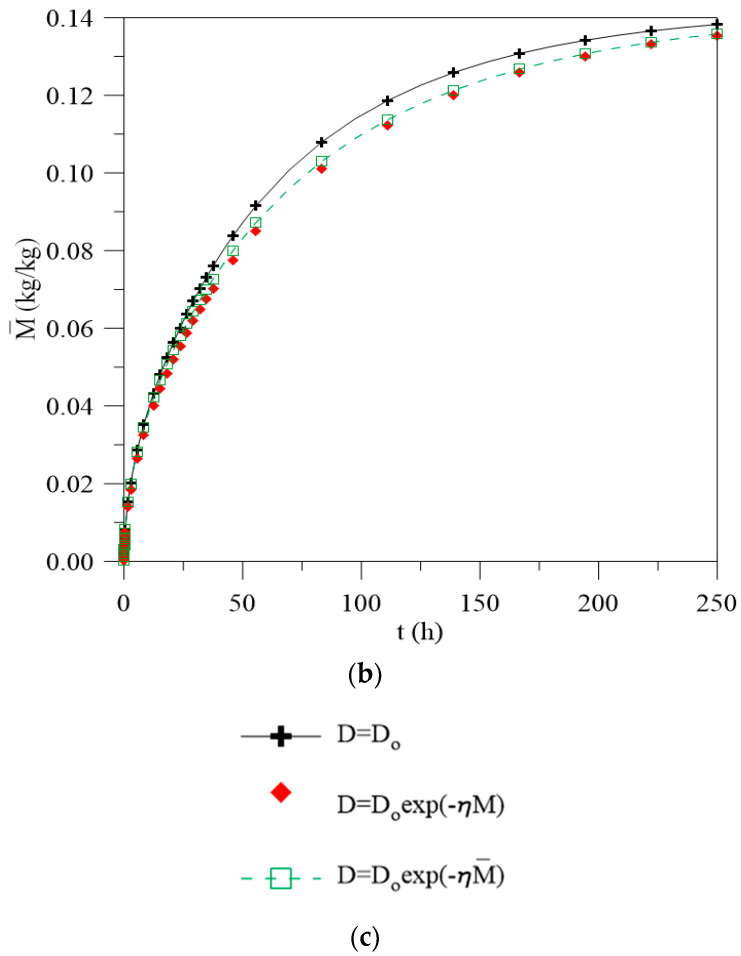
(**a**) Average moisture content of the vegetable fiber-reinforced polymer composite as a function of process time for different water mass diffusion coefficients (L1 = L2 = 0.100 m), (**b**) details of Figure 3a, (**c**) legend.

Figure 3 illustrates the average moisture content behavior in the composite as a function of time. The data were simulated considering the constant and variable mass diffusivity with the average and local moisture content, considering the same distances between the composite and the upper and lower water levels (L_1_ = 0.100 m and L_2_ = 0.100 m), as reported in Table 1. From the analysis of this figure, it is observed that water migration is quick in the initial times until a saturation condition is reached, where there is equilibrium with the outside environment. For the different approaches, there is also a clear difference between the moisture content values. The greatest difference between the cases evaluated is in the initial times, up to approximately 250 h. The larger differences in the values are observed at about t = 55 h; 4.73% for the cases where constant water mass diffusivity and variable with average moisture content are used and 7.19% for the cases where constant water mass diffusivity and variable with local moisture content are used. In addition, it appears that, when considering the constant water mass diffusivity, the material reaches hygroscopic balance more quickly, in about 750 h of the process (approximately 31 days), while for cases where this parameter changes as a function of average and local moisture content, this condition is reached in about 1111 h (approximately 46 days). This clearly indicates the effect of this variable on the water absorption process.

When the water mass diffusion coefficient is considered constant, the water absorption is faster, since it is independent of the water amount present inside the material. Whereas for the other cases, the water migration rate decreases as the average and local moisture content in the composite is increased. From a physical point of view, these approaches allow a different interpretation for each case. For the case of constant mass diffusivity, it is assumed that water penetrates at the same rate throughout the entire process. Then, different phenomena, such as fibers swelling and matrix plasticization, which can interfere in the moisture migration rate inside the material, are not considered. For other cases, where the water absorption rate depends on the moisture content retained in the material over time (average moisture) and locally (local moisture), it makes the predicted phenomenon even closer to what happens experimentally. Thus, depending on the type of material under study, considering constant water mass diffusivity throughout the entire process can lead to erroneous interpretations in relation to experimental phenomena and interfere in their applicability.

**Figure 4:**
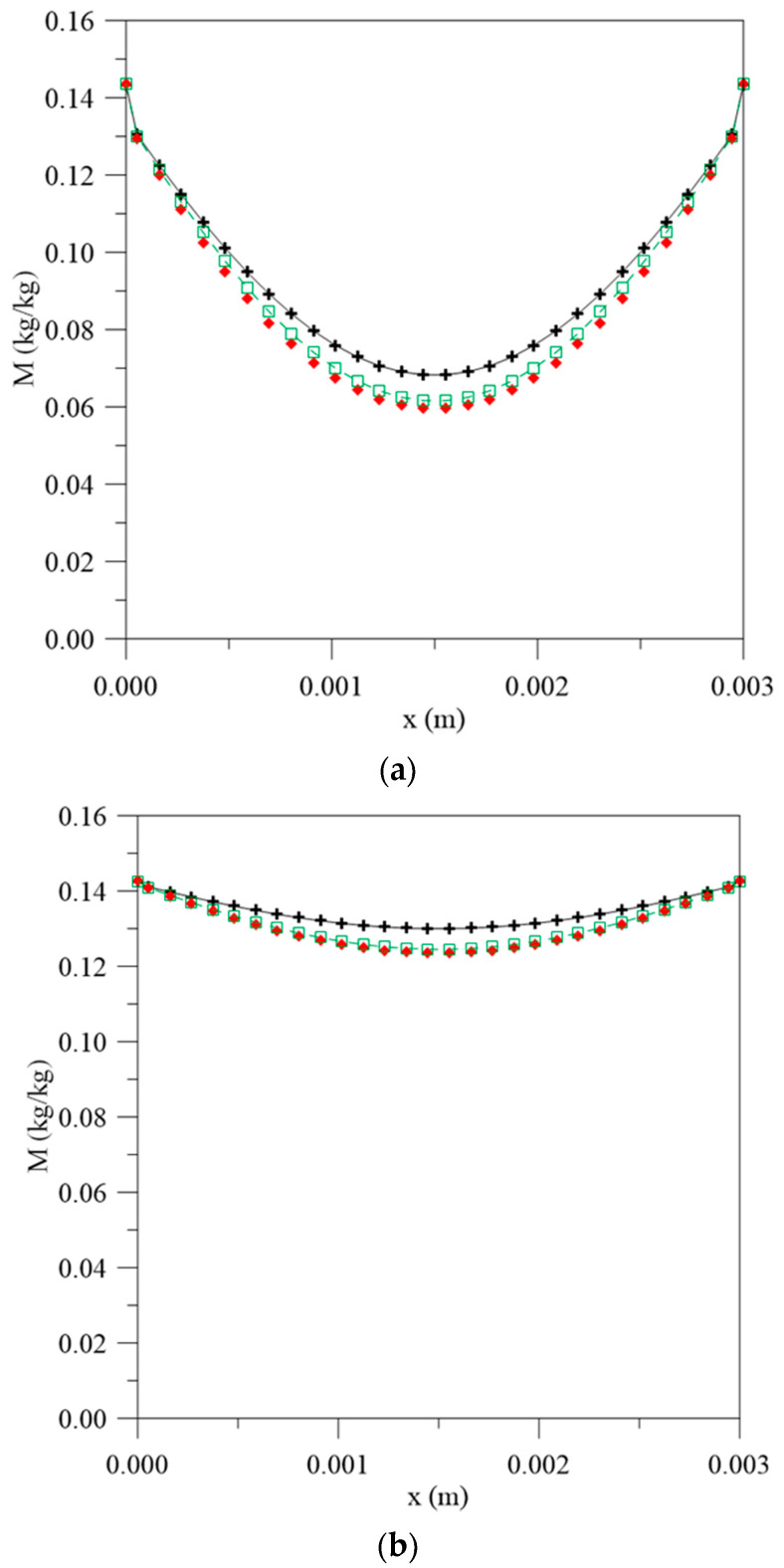

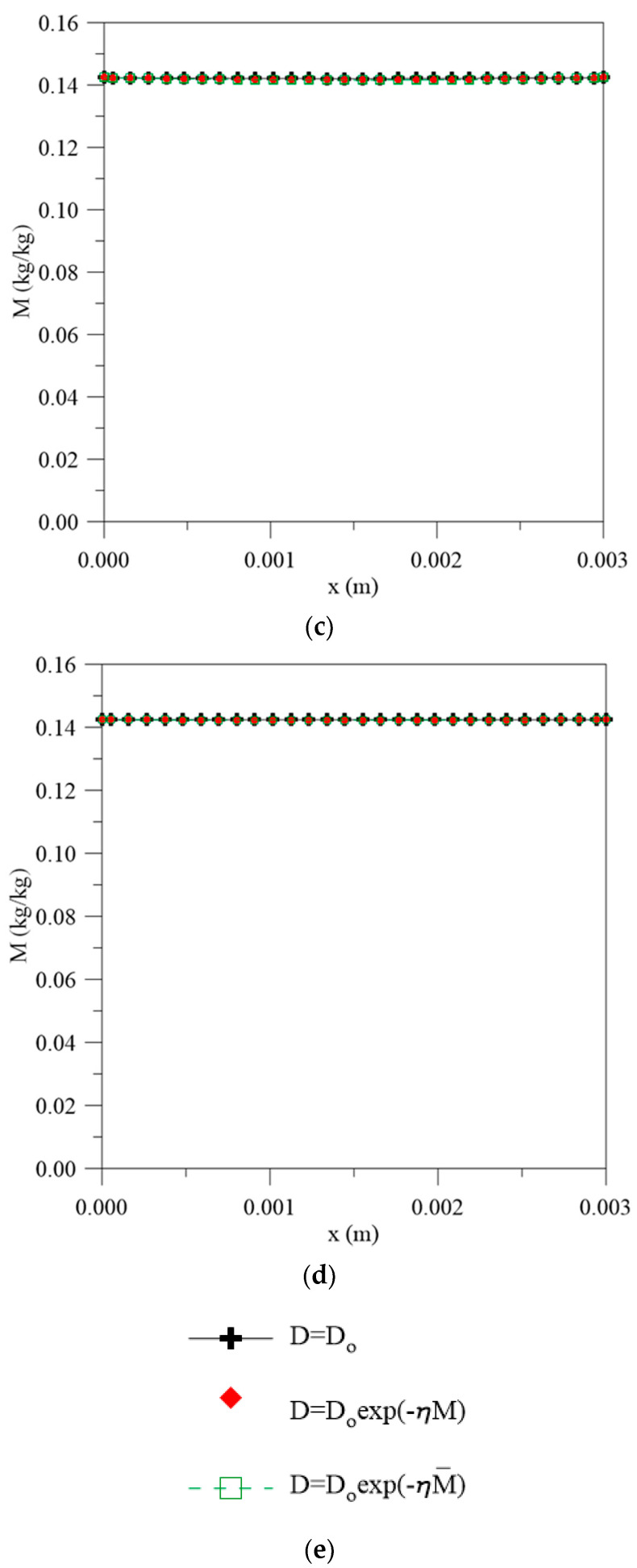
Local moisture content inside the vegetable fiber-reinforced polymer composite at different moments.(**a**) 56 h, (**b**) 195 h, (**c**) 500 h and (**d**) 1944 h for different water mass diffusion coefficients (L_1_ = L_2_ = 0.100 m), (**e**) legend.

Thus, in order to better understand the simulated phenomenon behavior, separate graphs were plotted of the free and entrapped water molecules concentration and local moisture content along the position and time. Figure 4 illustrates the local moisture content obtained from the sum of free and entrapped water molecules concentration along the material thickness at different process times for condition L_1_ = 0.100 m and L_2_ = 0.100 m. It is important to observe the influence of these parameters in the process; once the dimensional variables L_1_ and L_2_ assume the same values, there is symmetry in the absorption curve, that is, the simultaneous fluxes on both surfaces occur in the same way, as it presents the same water amount on the wall (container) available for diffusion.

From analyzing Figure 4, it can be seen that there is symmetry in the results obtained and that the highest values of the local moisture content are found on the material surface and the lowest in the center. The highest gradients of free water molecules concentration meet on the material surface and decrease towards the central region with increasing immersion time. Further, it is possible to observe that the largest differences in the value of this parameter are found in the initial times of the process (Figure 4a,b), with an error of 9.7% between the values obtained with constant mass diffusivity and variable with the average moisture content and 12.6% between the values obtained with constant mass diffusivity and variable with the local moisture content at a time of about 56 h. This is because the central regions of the composite are dried at the initial times of the process. Moreover, the free and entrapped water molecules concentration gradients and moisture uptake are greater for conditions of constant mass diffusivity, which leads to reaching the saturation condition faster than the other simulated cases.

**Figure 5:**
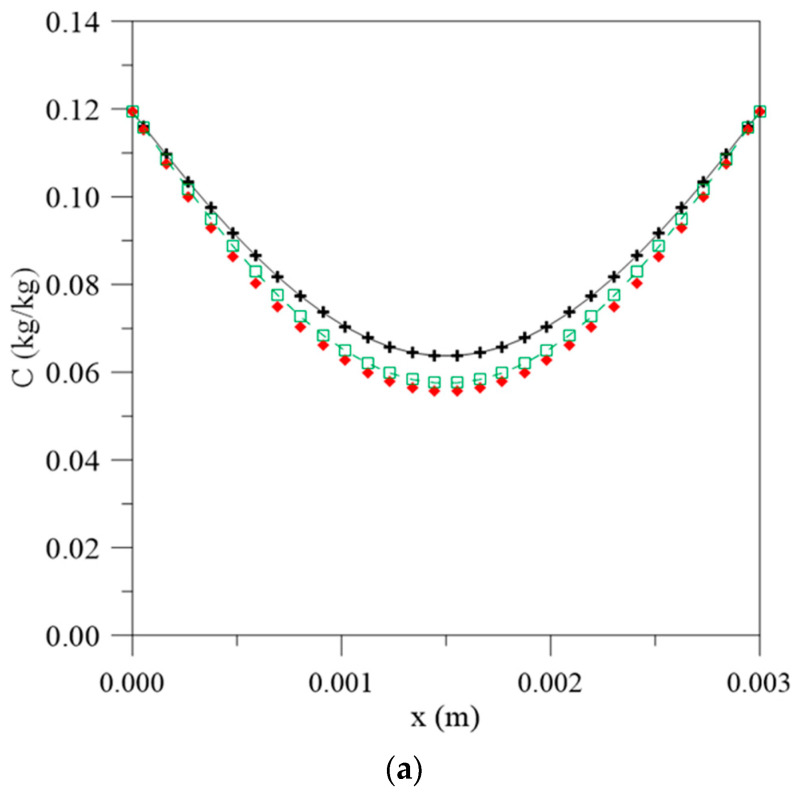

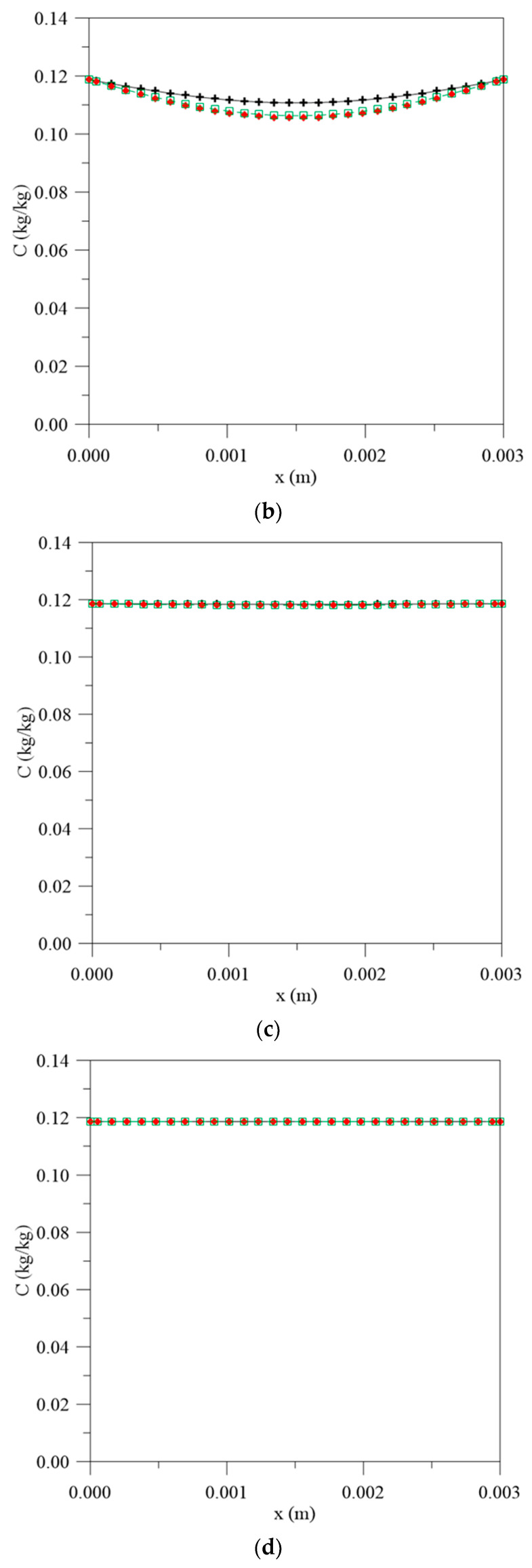

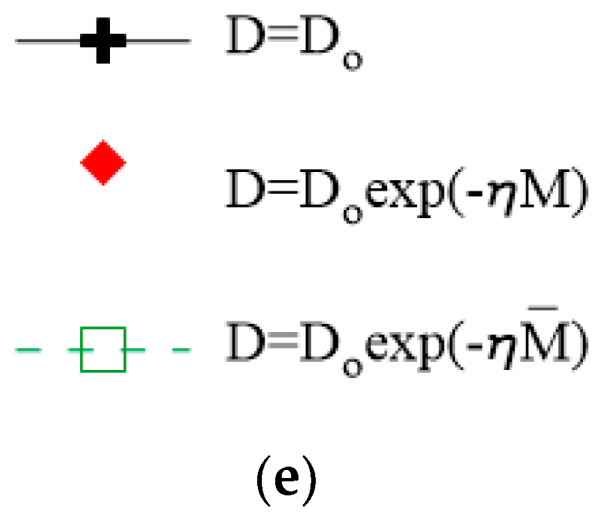
Free water molecules concentration inside the vegetable fiber-reinforced polymer composite at different moments. (**a**) 56 h, (**b**) 195 h, (**c**) 500 h and (**d**) 1944 h, for different water mass diffusion coefficients (L_1_ = L_2_ = 0.100 m), (**e**) legend.

**Figure 6:**
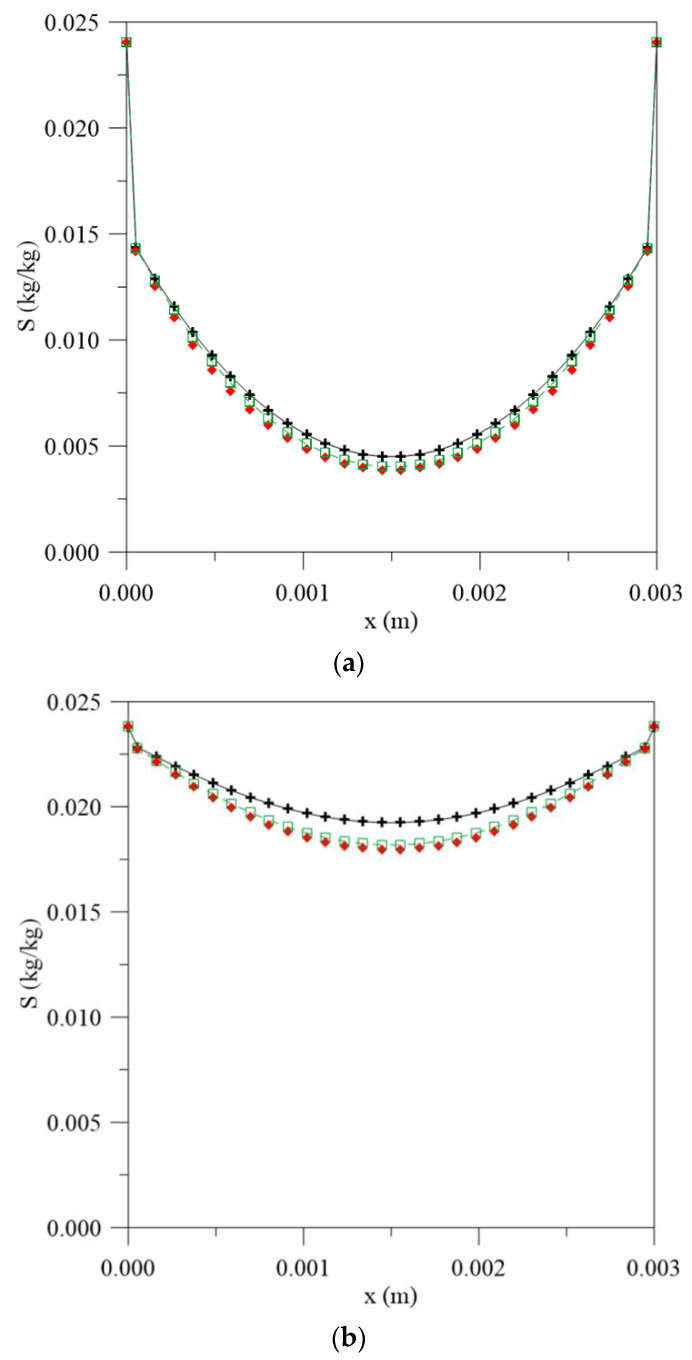

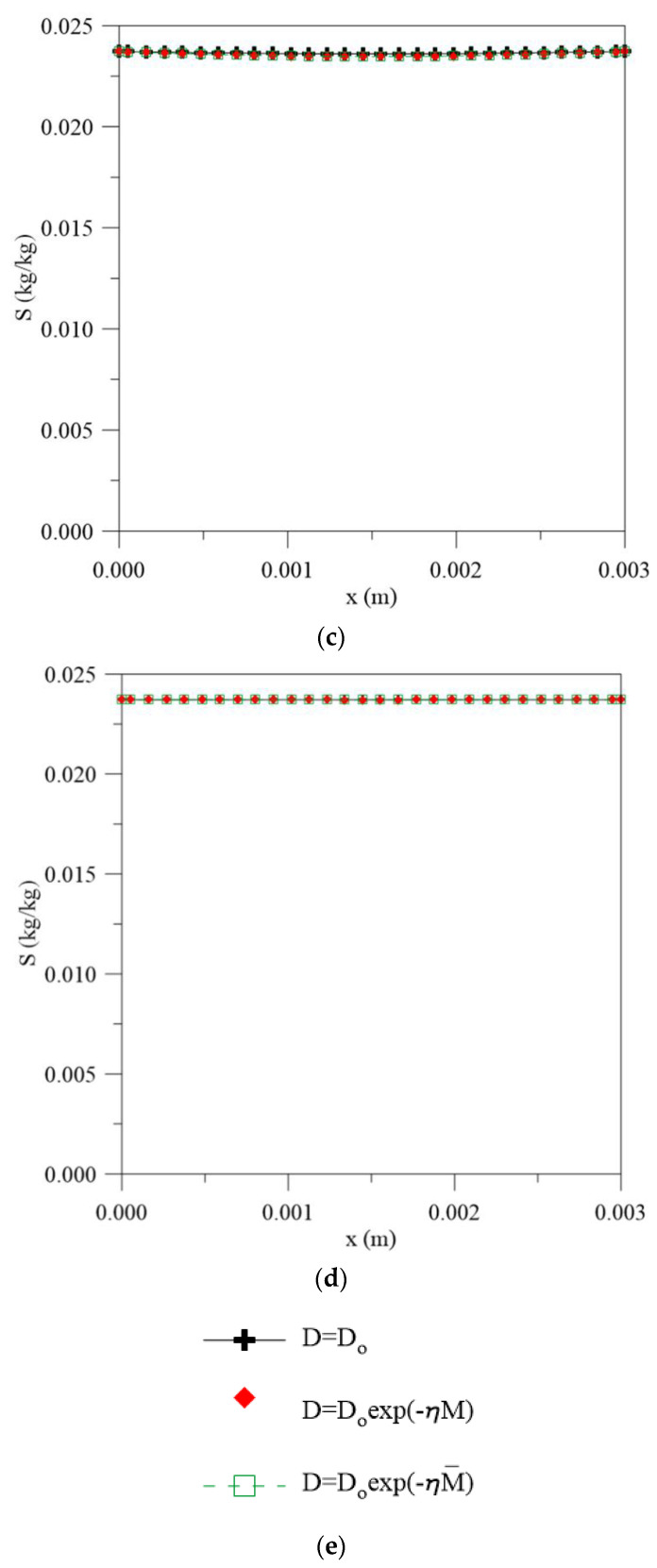
Entrapped water molecules concentration inside the vegetable fiber-reinforced polymer composite at different moments. (**a**) 56 h, (**b**) 195 h, (**c**) 500 h and (**d**) 1944 h, for different water mass diffusion coefficients (L_1_ = L_2_ = 0.100 m), (**e**) legend.

Figure 5 and Figure 6 illustrate the graphs for the free and entrapped water molecules concentration and the material thickness for different process times and water mass diffusivities. In Figure 5, it is possible to observe that the diffusion process is symmetrical on the material surfaces and the highest free water concentration gradients are found on the surface, with very close values for the three simulated cases, especially in the initial stages of the water absorption process. However, in the central regions, there is an increase in the difference between the values observed for the free water molecules concentration. When considering the constant diffusion rate, there is a greater concentration of free molecules for diffusion, so the process takes place with greater moisture migration velocity and tends to reach equilibrium condition more quickly with an increase in the exposure time.

For the concentration of entrapped water molecules (Figure 6), this difference is not as significant at the initial stages of the process (Figure 6a) and becomes more pronounced when the immersion time increases (Figure 6b) until hygroscopic equilibrium with the outside environment. This behavior delay can be explained when considering the terms “free” and “entrapped” as reversible and irreversible reactions with the polymer matrix. At the beginning of water sorption, moisture diffuses into the material as free molecules. As the immersion time increases, the polymer/water interaction reaches saturation and the sorption reaches a state of “quasi-equilibrium”; thus, the continuous exposure, there is a formation of an aggroupment of molecules (entrapped molecules) in the micro-voids present in the material structure until the saturation condition is reached [36].

**Figure 7:**
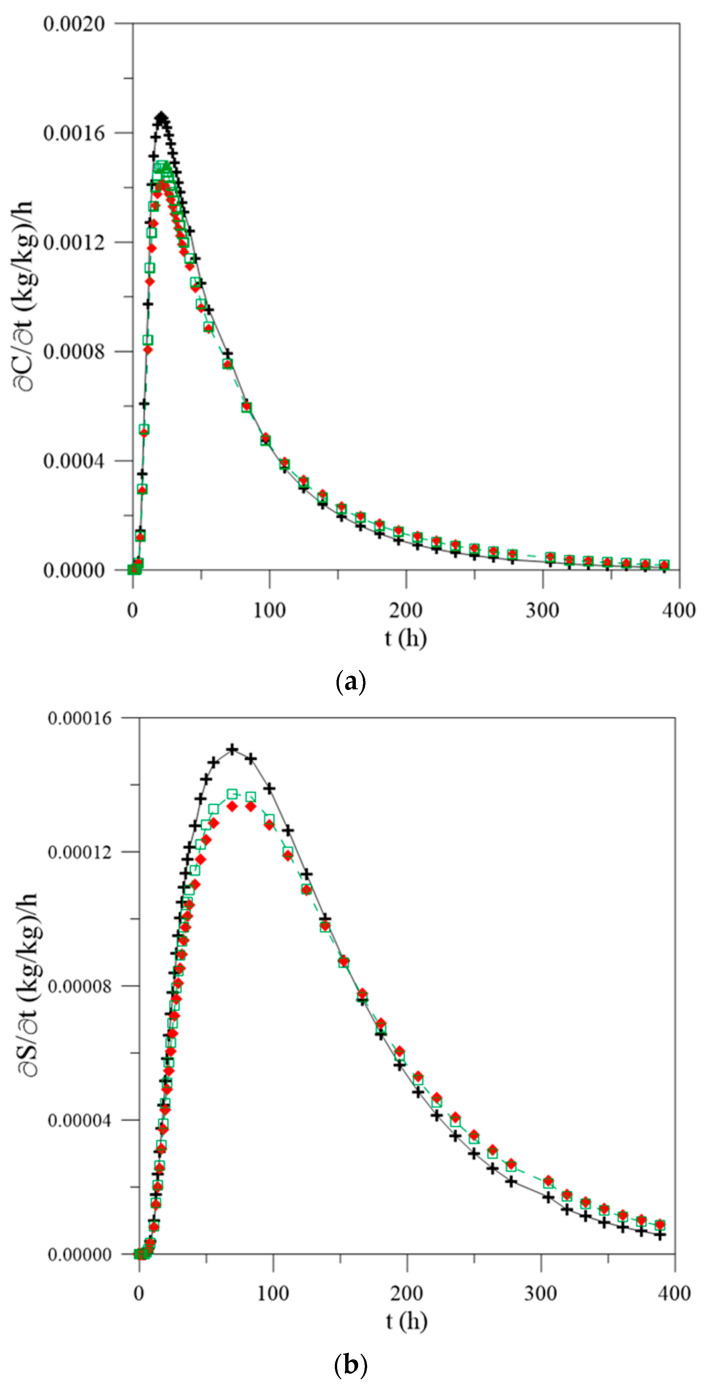

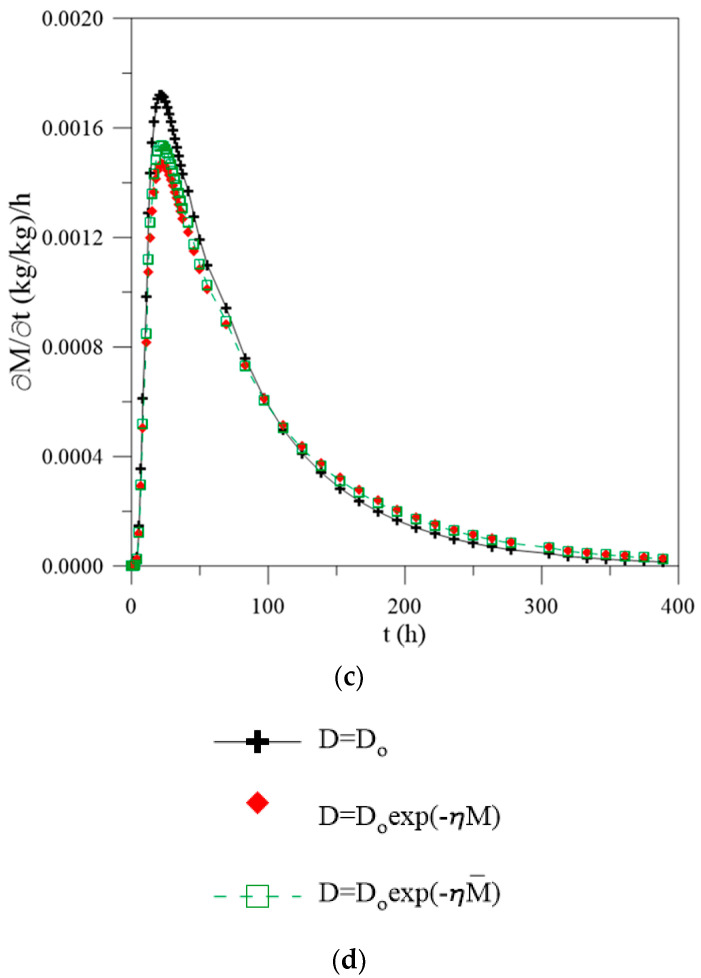
Temporal variation rate of (**a**) free water molecules concentration, (**b**) entrapped water molecules concentration and (**c**) local moisture content as a function of process time, in the center of the polymer composite and for different water mass diffusion coefficients (L_1_ = L_2_ = 0.100 m), (**d**) legend.

For a better understanding of the phenomena mentioned in this research, different graphs were plotted regarding the transient behavior of the variation rate (Figure 7) of the free (Figure 7a) and entrapped (Figure 7b) water molecules and local moisture content (Figure 7c). From the analysis of this figure, it is possible to observe that the humidification process is fast in the first hours of the process, assuming an increasing rate until reaching a maximum absorption point. For the three simulated cases (different mass diffusion coefficients), the material reaches this point in approximately 24 h; however, they present different values for the moisture content 17%, 15% and 14% for the constant diffusivity and vary with the average and local moisture content, respectively, until that saturation is reached. The free water molecules concentration reaches a saturation peak at 22 h elapsed time for constant water mass diffusivity, with a value of 0.021 (kg/kg)/h, whereas, for water mass diffusivity varying with the average and local moisture content, peaks were verified at 22 h with the value of 0.0189 (kg/kg)/h and 24 h with 0.0200 (kg/kg)/h, respectively.

For the concentration of entrapped water molecules, when the diffusivity is considered to be constant or changing with the average moisture content, the absorption peak is reached in approximately 83 h with values of 0.0086 (kg/kg)/h and 0.0078 (kg/kg)/h, respectively, whereas, for the case where the mass diffusivity is a function of the local moisture content, the peak is observed at about 97 h, reaching a value of 0.0093 (kg/kg)/h. These values justify the difference in behaviors observed in Figure 5 and Figure 6 and agreed with the theories reported in the literature, since free water has the ability to move independently along the material, while the entrapped molecules are restricted to polar groups present in the structure of the composite. In addition, considering that the constant water mass diffusivity implies major differences in the real values for the parameters, it is more coherent, from the physical point of view to consider the water mass diffusivity varying with the local moisture content.

**Figure 8:**
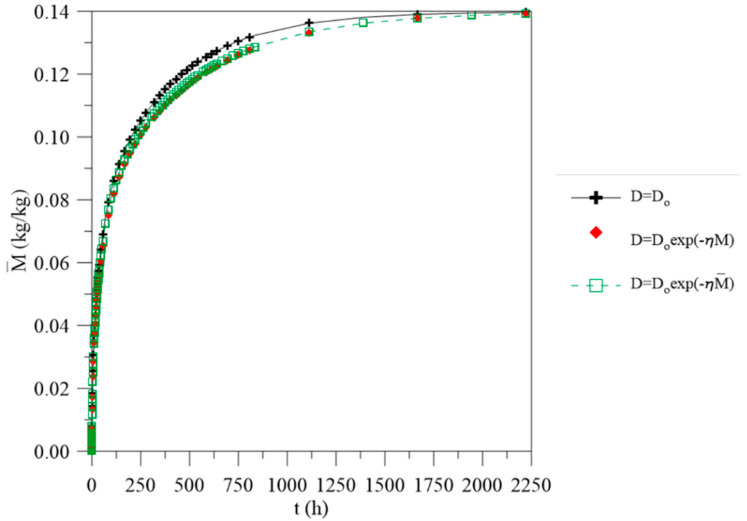
Average moisture content of the vegetable fiber-reinforced polymer composite as a function of process time for different water mass diffusion coefficients (L_1_ = 0.100 m and L_2_ = 0.001 m).

Figure 8 illustrates the graph obtained for the average moisture content in the material as a function of the process’s time, considering the constant water mass diffusivity and varying with the average and local moisture content, when the distances between the composite and the container are different (L_1_ = 0.100 m and L_2_ = 0.001 m). For this new physical situation, changes in the water absorption kinetics are observed due to the difference in the geometric process parameters. The change in distance from the bottom surface of the composite to the bottom of the container causes changes in the water absorption kinetics, since this phenomenon is dependent on the contact area between the water and the material. For the initial stages of process, there is no difference in the average moisture content estimated when using the different water mass diffusivities, which is different from the behavior shown in Figure 3. For this new case, the differences are more significant after about 500 h of the process, where a difference of 3.23% is observed between the values of the average moisture content predicted when water mass diffusivity is considered to be constant and when it varies as a function of the average moisture content, and a difference of 4.48% is observed when this parameter changes with the local moisture content.

In addition, in comparison to Figure 3, the material tends towards an equilibrium state in approximately 375 h; however, when the diffusivity is constant, the material retains a greater moisture amount. For this instant of time, for example, the values for the moisture content are 0.1414 kg/kg, 0.1404 kg/kg and 0.1405 kg/kg, for the cases in which diffusivities are constant, are a function of the local moisture and are a function of the average moisture, respectively. For cases where there is a change in geometric parameters (Figure 8), the material tends to reach equilibrium in longer times, after about 1625 h. Compared with the previous case, at 375 h, it is observed the values for the moisture content of 0.1150 kg/kg, 0.1099 kg/kg and 0.1118 kg/kg, for the different cases, respectively.

**Figure 9:**
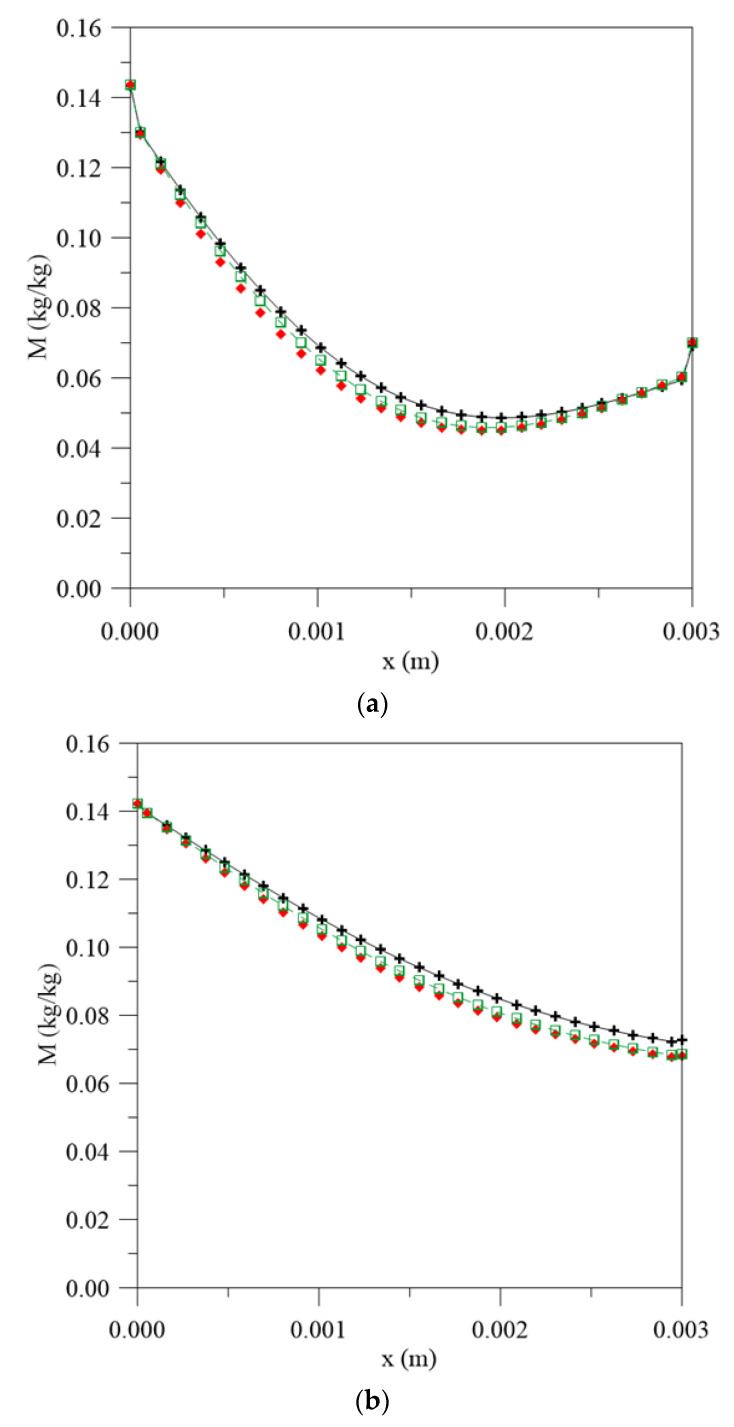

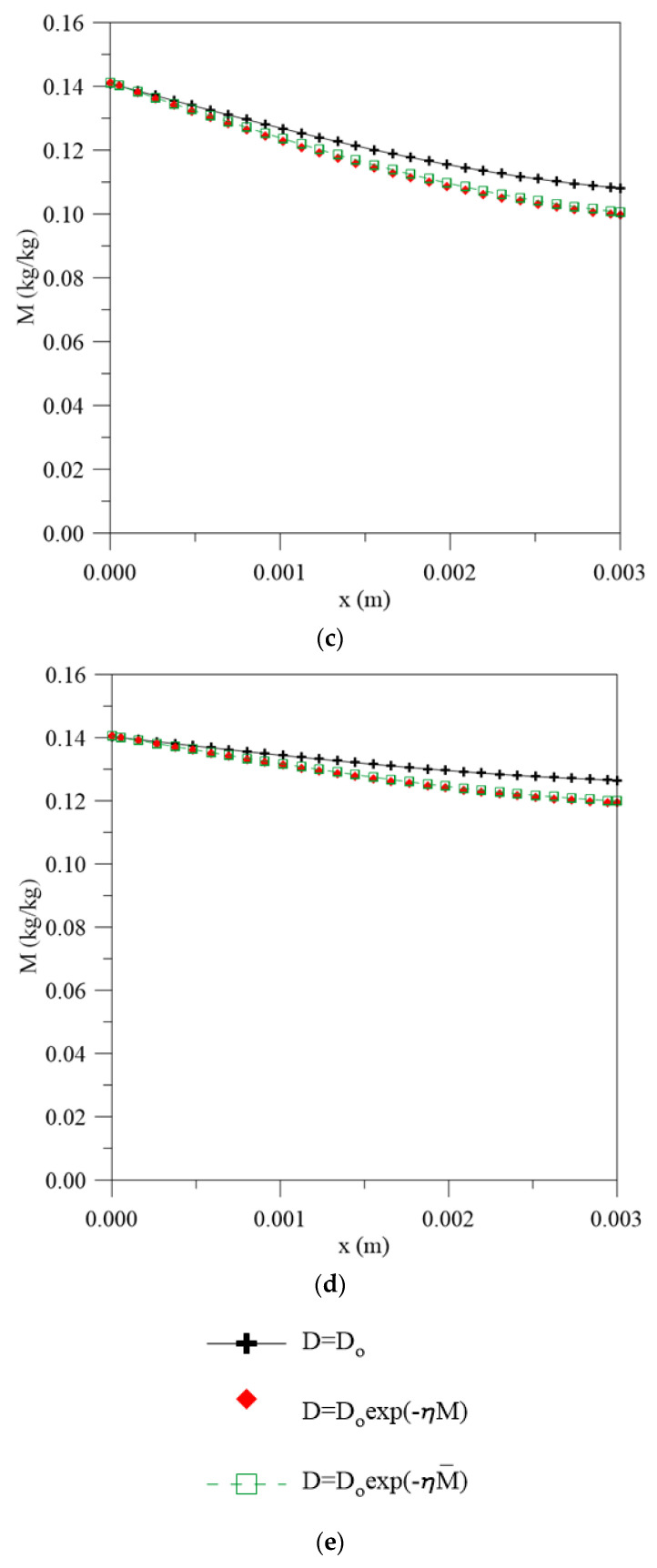
Local moisture content inside the vegetable fiber-reinforced polymer composite at different moments. (**a**) 56 h, (**b**) 195 h, (**c**) 500 h and (**d**) 1944 h, for different water mass diffusion coefficients (L_1_ = 0.100 m and L_2_ = 0.001 m), (**e**) legend.

Figure 9 illustrates the local moisture content inside the vegetable-fiber-reinforced polymer composite for the case when L_1_ = 0.100 m and L_2_ = 0.001 m at different moments of the process. In this figure, it can be seen that each curve assumes an asymmetric behavior with the highest moisture content gradients in the regions where the composite-water level distance is greatest. In the initial process times, small differences are observed between the different values of the moisture content estimated by different mass diffusion coefficients used in the simulations, especially in the regions close to the center of the material. With an increase in immersion time, the difference becomes more pronounced in the regions close to the surface, reaching an error of 7.0% between the predicted values, considering constant mass diffusion coefficient and variable with the average moisture content, and 7.8% between the values when using constant mass diffusion coefficient and variable with the local moisture content, at about 500 h. This behavior can be explained due to the water amount available for diffusion, since this process occurs preferably in the region where the distance between the composite surface and water level assumes a greater value.

**Figure 10:**
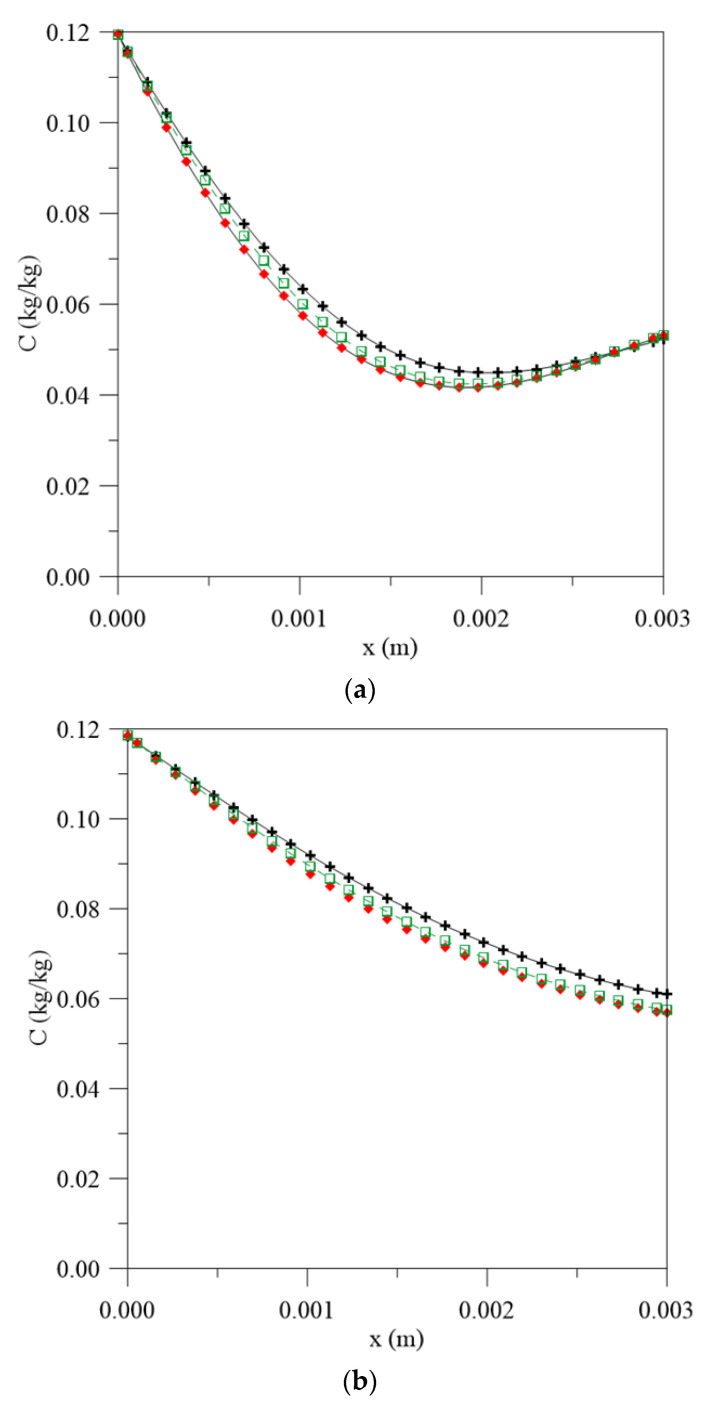

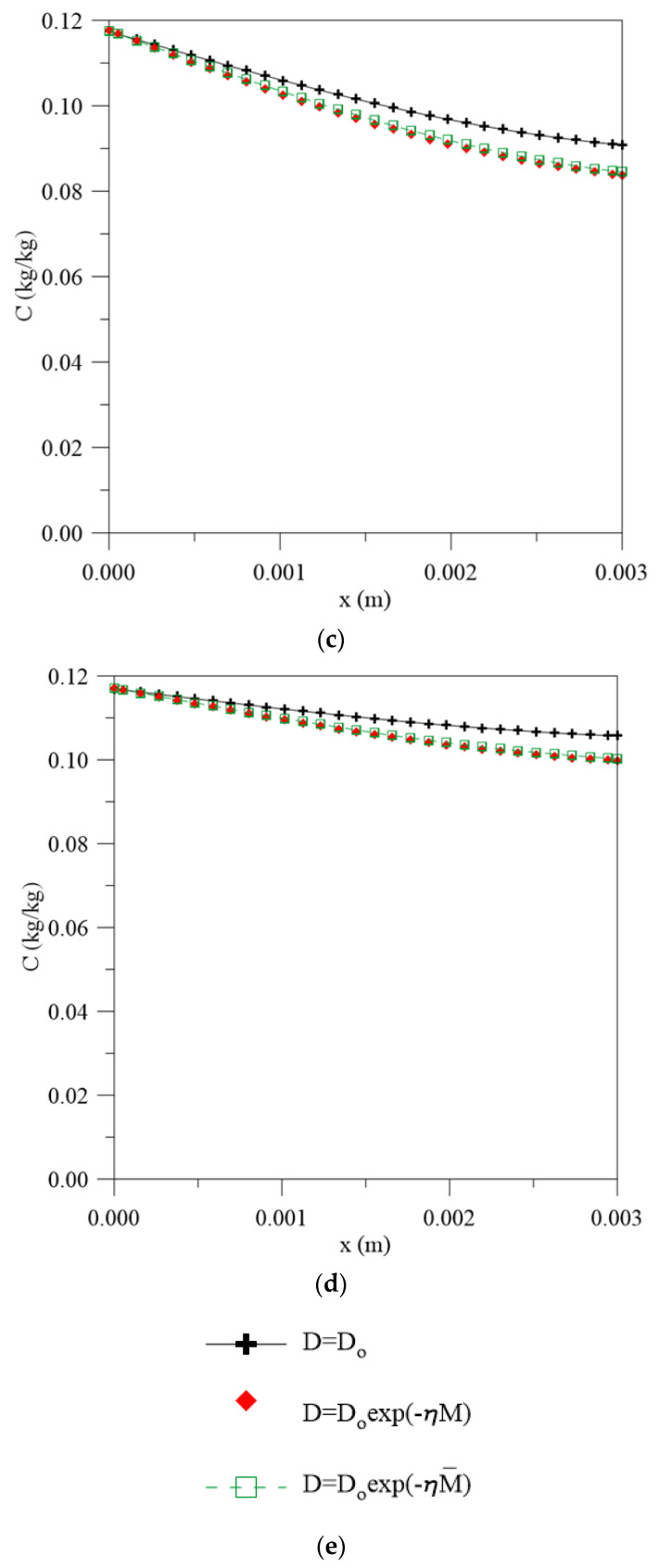
Free water molecules concentration inside the vegetable fiber-reinforced polymer composite at different moments. (**a**) 56 h, (**b**) 195 h, (**c**) 500 h and (**d**) 1944 h, for different water mass diffusion coefficients (L_1_ = 0.100 m and L_2_ = 0.001 m), (**e**) legend.

**Figure 11:**
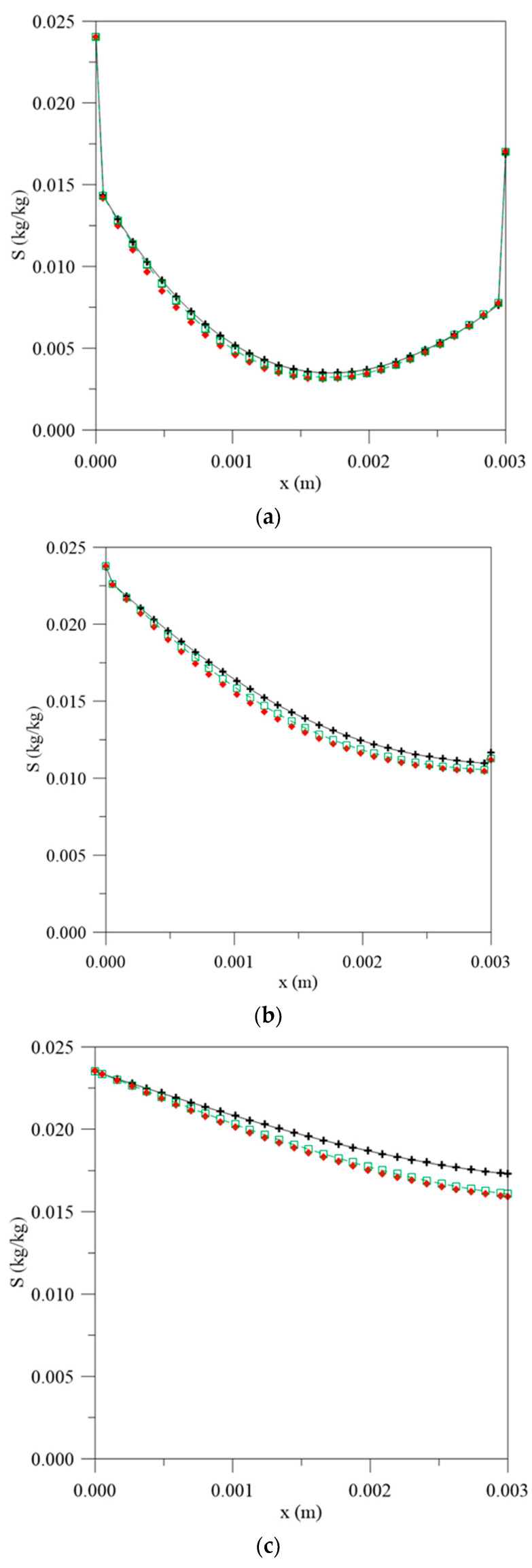

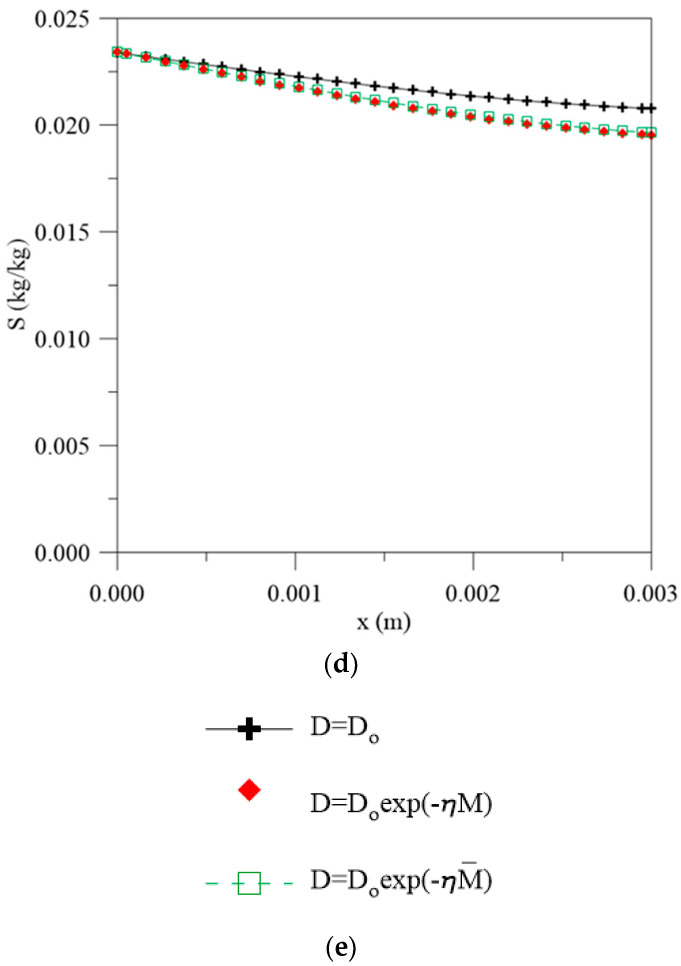
Entrapped water molecules concentration inside the vegetable fiber-reinforced polymer composite at different moments. (**a**) 56 h, (**b**) 195 h, (**c**) 500 h and (**d**) 1944 h, for different water mass diffusion coefficients (L_1_ = 0.100 m and L_2_ = 0.001 m), (**e**) legend.

The free and entrapped water molecules profiles inside the vegetable-fiber-reinforced polymer composite are illustrated in Figure 10 and Figure 11, respectively, for different process times, for the case of L_1_ = 0.100 m and L_2_ = 0.001 m. Upon analyzing Figure 10, it is possible to verify that the highest free water concentration gradients are found in the region where the L assumes the highest value. For the initial times (Figure 10a), the largest differences are observed in the central regions of the composite, assuming a value of approximately 6.8% between the values obtained when using the constant diffusion coefficient and varying with the average moisture content and 10% between the values when using the constant diffusion coefficient and varying with the local moisture content. These values tend to change with both the immersion time and L value. With continuous exposure (Figure 10d), these differences in the predicted values of the moisture content tend to decrease to about 5.1% and 5.6%, respectively, being predominant on the surface where L assumes a lower value, due to the smaller amount of water available for diffusion.

Figure 11 illustrates the entrapped water molecules profile inside the vegetable-fiber-reinforced polymer composite along the material thickness, at different process times and mass diffusion coefficients, for the case when L_1_ = 0.100 m and L_2_ = 0.001 m. From this figure, it is possible to observe that the greatest differences are found in the longer immersion times (Figure 11c,d), reaching about 7.1% between the values obtained when using the constant diffusion coefficient and varying with the average moisture content, and 8.0% between the values obtained when using the constant diffusion coefficient and varying with the local moisture content. 

In contrast, as observed in Figure 6, the greatest differences are on the surface where L assumes a small value, instead of the central regions of the material, which can be justified by the process asymmetry due to the change in the distance between the level water and the composite surface.

**Figure 12:**
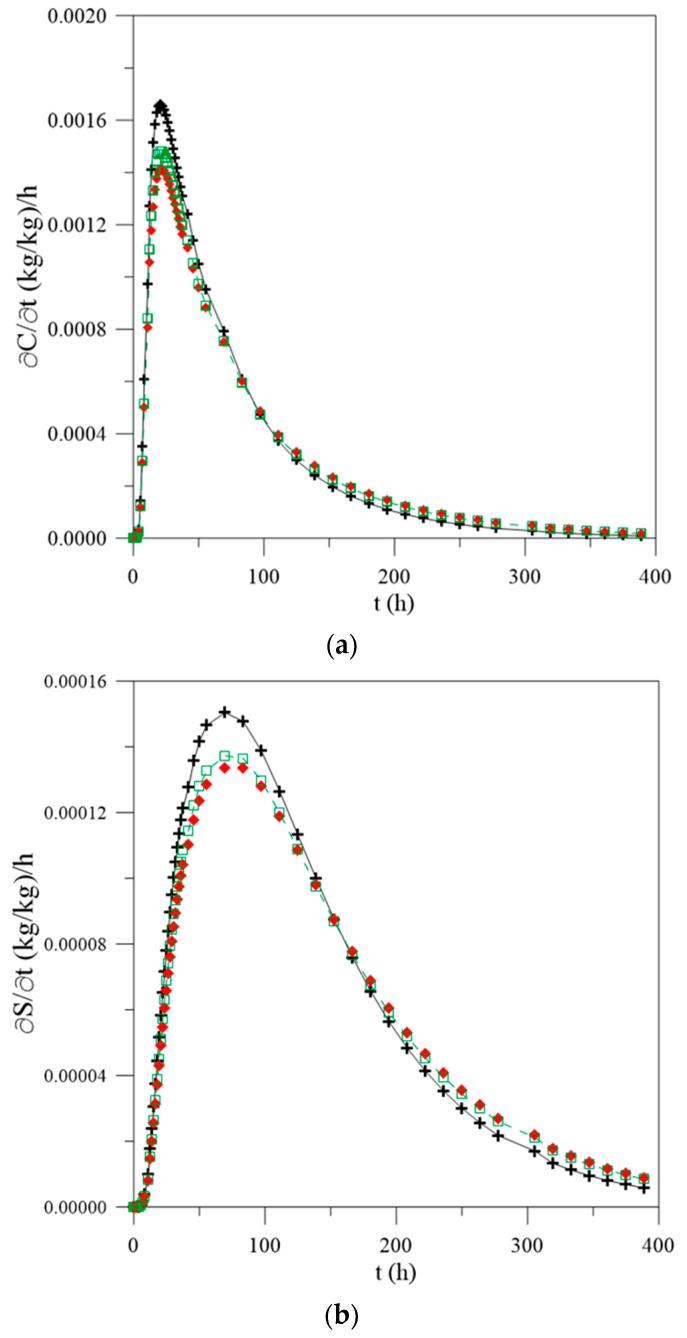

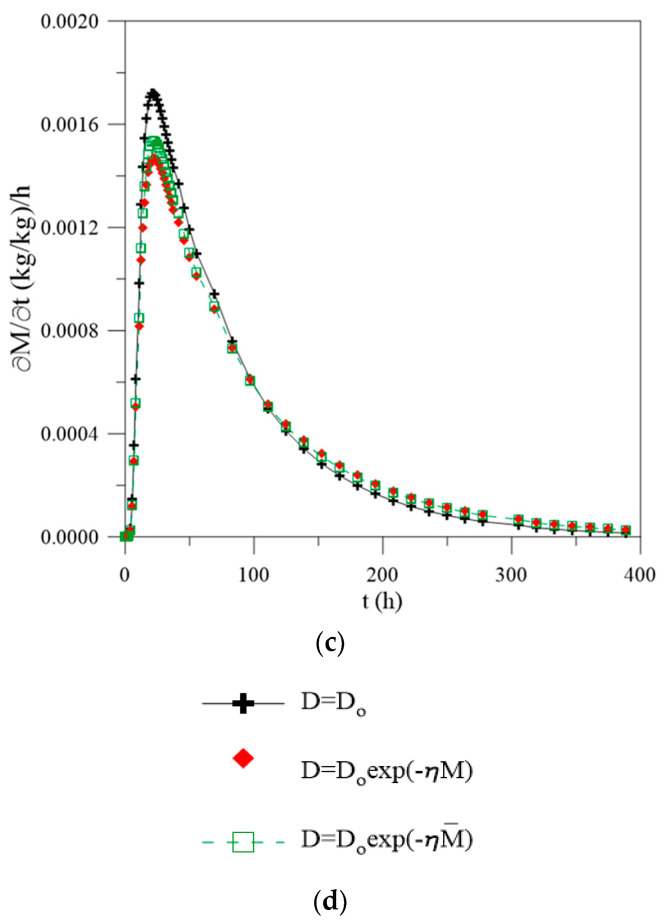
Temporal variation rate of (**a**) concentration of free water molecules, (**b**) concentration of entrapped water molecules and (**c**) local moisture content as a function of process time, in the center of the polymer composite and for different water mass diffusion coefficients (L_1_ = 0.100 m and L_2_ = 0.001 m), (**d**) legend.

Figure 12 illustrates the transient behavior of the rate of variation of the free (Figure 12a) and entrapped (Figure 12b) water molecules concentration and local moisture content (Figure 12c) as a function of the time of the process, for the case where L_1_ = 0.100 m and L_2_ = 0.001 m. For purposes of comparison with Figure 7, when the geometric values of L_1_ and L_2_ are equal, the maximum absorption rate values are shown in Table 2. For the case in which L_1_ = L_2_ = 0.100 m, it is observed that the maximum absorption point occurred at approximately 24 h for all water mass diffusion coefficients used; however, for the case in which L_1_ = 0.100 m and L = 0.001 m, the peak occurred at 20.8 h for constant diffusivity and in 22.2 h for other cases.

For cases where the water mass diffusivity is constant, values for the moisture content in the material are approximately 16% higher when compared with obtained considering water mass diffusivity variable with average moisture content, and the material reaches an absorption peak more quickly, at approximately 21 h, regardless of the change in geometric parameters. Further, for the other case, there is a small change in this behavior, namely about 6% difference in the estimated values considering water mass diffusivity variable with local moisture content. Thus, it can be inferred that estimating the materials’ properties considering mass diffusion coefficient varying with the local moisture content, it is possible to obtain values of these parameters with minor errors in relation to the real experiments and more coherent from the physical point of view.

As a general comment, we state the following characteristics and potential of the proposed mathematical formulation:

(a) It can be applied to different vegetable-fiber-reinforced polymer composite.
(b) There are restrictions for the use of vegetable fiber. This way, any vegetable fiber can be used as reinforcement, for example, sisal, ramie, hemp, kenaf, jute, caroá, curauá and many others. However, it is recommended to use treated fiber in order to reduce the effects of water absorption in the composite, such as weak adhesion between fiber surface and matrix surface, which strongly affect the mechanical performance of this material.
(c) There are no restrictions for the use of polymer matrix. However, it is recommended to use unsaturated thermoset resin, for example, polyester. This resin is most appropriate for this purpose, due to its low cost and satisfactory mechanical properties.
(d) Since the one-dimensional approach was proposed, restrictions to sample shape are not applied. Results are only dependent on the sample thickness.
(e) Since homogeneous material is assumed in the proposed model, fiber arrangement in the matrix must be directed for this consideration. This way, it is recommended to use a random arrangement of short fiber in the resin.

Finally, it is very important to speak about industrial applications of the vegetable-fiber-reinforced polymer composite. In the present day, vegetable-fiber-reinforced polymer composite materials have been used in different sectors such as automotive, building, transportation, consumer goods, sports equipment, design equipment and many more, especially when moderate mechanical performance is expected. This way, the study complement reported researches in this area, in order to assist engineers, specialists, industrials, academics, etc. in making decisions about a specific polymer composite in operation under a moist environment.

## 4. Conclusions

In this research, mathematical modeling based on Langmuir’s model was presented, to predict the water absorption behavior in polymer composite materials considering both constant and variable mass diffusion coefficients as a function of the average and local moisture content. From the numerical results obtained, it can be concluded that:

(a) The water layer thickness influences the water absorption kinetics. When the values of the water layer thickness are equal, a difference is observed between the cases evaluated at the initial times, up to approximately 250 h. For the cases where the values of the water layer thickness are different, there is a change in the water absorption kinetics, due to the smaller contact area between the water and the composite; the most significant differences in values of the average moisture content were observed after about 250 h of the process.
(b) When the mass diffusion coefficient is considered constant, the water absorption process is faster, whereas, for cases where the mass diffusion coefficient varies with the average and local moisture content, the water migration velocity changes with an increasing average and local moisture content inside the composite.
(c) The largest concentration gradients of free and entrapped water molecules are found on the material surface and decrease towards the central region, and with increasing immersion time. The biggest differences between the values obtained from these parameters for each condition of the mass diffusion coefficient (constant and variable) are found in the initial stages of the process.
(d) Considering constant water mass diffusion coefficient and equal water layer thickness at all faces of the polymer composite, a difference of about 16% is obtained in the moisture content values in comparison with those obtained when considering the mass diffusivity varying with the local moisture content at time t = 22.2 h. For the case where the geometric parameters are different, this difference is about 6%; however, the material reaches maximum absorption more quickly at t = 20.8 h.

In general, this research proposes a new approach for the water absorption behavior in polymer composite materials when considering the mass diffusivity varying with both the average and local moisture content, thus providing a greater understanding of the physical and mathematician point of view of the phenomenon and how it occurs in these materials.

## Figures and Tables

**Table 1:** Operational conditions used in the computational simulation.

Case	Parameter							
	Water	Composite						
	Te (°C)	To (°C)	2a (m)	μ (10^−6^ s^−1^)	λ (10^−6^ s^−1^)	D (m^2^ s^−1^)		
						Constant	Variable ^1^	
						Do	f(M)	f(M)
							η	η
L_1_ = 0.100 m L_2_ = 0.100 m	25	25	0.003	5	1	$5\times 10^{-12}$	0	0
	25	25	0.003	5	1	$5\times 10^{-12}$	2	0
	25	25	0.003	5	1	$5\times 10^{-12}$	0	2
L_1_ = 0.100 m L_2_ = 0.001 m	25	25	0.003	5	1	$5\times 10^{-12}$	0	0
	25	25	0.003	5	1	$5\times 10^{-12}$	2	0
	25	25	0.003	5	1	$5\times 10^{-12}$	0	2

^1^ Do = 5 × 10^−12^.

**Table 2:** Concentration values of C, S and M and their respective absorption rate in the center of the composite, for different operating conditions.

Case	D	t (h)	C (kg/kg)	∂C/∂t	t (h)	S (kg/kg)	∂S/∂t	t (h)	M (kg/kg)	∂M/∂t
L_1_ = 0.100 m L_2_ = 0.100 m	Constant	20.8	0.019	0.00166	83.3	0.008	0.000148	23.6	0.0240	0.00171
	f(M)	22.2	0.018	0.00141	97.2	0.009	0.000128	23.6	0.0200	0.00147
	$f\left(\bar{M}\right)$	22.2	0.016	0.00148	83.3	0.008	0.000136	23.6	0.0210	0.00153
L_1_ = 0.100 m L_2_ = 0.001 m	Constant	20.8	0.017	0.00140	69.4	0.0053	0.00011	20.8	0.0173	0.00145
	f(M)	20.8	0.014	0.00123	83.3	0.0062	0.00010	22.2	0.0167	0.00127
	$f\left(\bar{M}\right)$	22.2	0.017	0.0127	83.3	0.0063	0.00010	22.2	0.0174	0.00132

## Data Availability

The study did not report any data.
